# Diversity and structure of the deep-sea sponge microbiome in the equatorial Atlantic Ocean

**DOI:** 10.1099/mic.0.001478

**Published:** 2024-07-29

**Authors:** Sam E. Williams, Gilda Varliero, Miguel Lurgi, James E.M. Stach, Paul R. Race, Paul Curnow

**Affiliations:** 1 School of Biochemistry, University of Bristol, University Walk, Bristol, BS8 1TD, UK; 2 Novo Nordisk Foundation Center for Biosustainability, Technical University of Denmark, Søltofts Plads, Building 220, 2800 Kgs., Lyngby, Denmark; 3 Rhizosphere Processes Group, Swiss Federal Institute for Forest, Snow and Landscape Research (WSL), Birmensdorf, Switzerland; 4 Department of Biosciences, Swansea University, Singleton Park, Swansea, SA2 8PP, UK; 5 School of Natural and Environmental Sciences, Newcastle University, Newcastle upon Tyne, NE1 7RU, UK

**Keywords:** 16S rRNA gene analysis, deep sea, metataxonomics, microbiome, Porifera, sponge

## Abstract

Sponges (phylum Porifera) harbour specific microbial communities that drive the ecology and evolution of the host. Understanding the structure and dynamics of these communities is emerging as a primary focus in marine microbial ecology research. Much of the work to date has focused on sponges from warm and shallow coastal waters, while sponges from the deep ocean remain less well studied. Here, we present a metataxonomic analysis of the microbial consortia associated with 23 individual deep-sea sponges. We identify a high abundance of archaea relative to bacteria across these communities, with certain sponge microbiomes comprising more than 90 % archaea. Specifically, the archaeal family Nitrosopumilaceae is prolific, comprising over 99 % of all archaeal reads. Our analysis revealed that sponge microbial communities reflect the host sponge phylogeny, indicating a key role for host taxonomy in defining microbiome composition. Our work confirms the contribution of both evolutionary and environmental processes to the composition of microbial communities in deep-sea sponges.

**Impact statement**

The deep ocean is the largest biome on Earth, accounting for >90 % of the planet’s marine environment. Despite this, it remains a largely unexplored ecosystem, with less than 0.01 % of the deep seafloor having been quantitatively sampled. Deep-sea sponges are ancient metazoans that harbour complex microbial communities and much still remains to be learnt about the composition and diversity of these unique microbiomes. To address this, here, we report a metataxonomic analysis of the microbial consortia associated with 23 deep-sea sponges from the equatorial Atlantic Ocean. Our findings reveal intricate, species-specific microbial communities dominated by ammonia-oxidizing archaea. This study highlights the significant role sponges play in shaping microbial consortia, providing new insights into deep-sea ecosystem dynamics. Importantly, our findings provide a scientific basis for understanding the evolutionary relationships between sponges and their symbiotic microorganisms.

## Introduction

Sea sponges (phylum Porifera) live in intimate symbiotic relationships with complex microbial communities [1,3]. Microbial abundance within sponge tissue can be orders of magnitude greater than the surrounding seawater [4]. Sponge microbiotas are generally dominated by Pseudomonadota and a few other core phyla [5], but at least 30 other variable phyla are commonly identified in studies of metataxonomic communities. Microbial sponge symbionts are believed to play a vital role in nutrient cycling for the sponge host, including carbon fixation, B vitamin synthesis, sulphite oxidation and nitrogen metabolism [6,7]. These communities exhibit unusual features such as the presence of the sponge-associated phyla known as Poribacteria, which are only rarely observed living outside the sponge hosts [8,9]. Large-scale comparative surveys have confirmed that microbial communities are relatively stable within, but vary greatly in richness and diversity across, sponge species [8,11]. This species dependence suggests that particular microorganisms might be selected for by sponge–microbe or microbe–microbe interactions. However, these relationships might be modulated by other biotic and abiotic factors; for example, water temperature appears to have a major influence on microbial community structure inside hosts [9,12].

Much of our current knowledge of sponge-associated microbes is derived from samples collected in relatively shallow coastal waters [5]. There are fewer studies investigating sponges that inhabit the deep ocean [13], at least in part due to the difficulty in obtaining such samples. Sponge communities from mesopelagic and bathypelagic depths might be expected to differ from their shallow-water relatives, given the drastically different ambient conditions – cold, dark and elevated hydrostatic pressure – and the consequent changes to the microbial life in the seawater around the sponge. Current evidence suggests that deep-water sponge-associated bacterial communities remain species-specific and that sponges with both high and low microbial abundance (HMA and LMA, respectively) are found at these depths [10,14, 15]. Perhaps the major differences in the community structure of deep-sea sponges are the general loss of Cyanobacteriota and a lower occurrence of Poribacteria, as well as the increased abundance of archaea [14,16]. Archaea are found to be more abundant than bacteria in some deep-water sponges and are almost entirely dominated by the phylum Thermoproteota, specifically the family Nitrosopumilaceae [16,17]. As with shallow-water sponges, the precise composition of deep-water sponge communities reflects the complex relationship between evolutionary and environmental factors [10,18].

The recent shift towards whole genome-based taxonomy has significantly revised the microbial tree of life [19]. Traditional taxonomic assignment via outdated 16S ribosomal RNA (rRNA) databases fails to account for this revision and performs poorly in accurately classifying standardized mock communities [20]. The newly developed Greengenes2 unifies whole genome taxonomy and 16S rRNA, offers improved accurate taxonomic assignment and has not yet been applied to the sponge microbiome [21]. In addition to their fundamental role in sponge ecology, the microbial communities of deep-sea sponges are also of increasing interest as a resource for novel therapeutics. Sponge symbionts are well established as a fertile repository of bioactive natural products [22], and the diverse composition of sponge microbiota has the potential for high and novel functional diversity [23]. The microbial consortia of deep-sea sponges remain largely untapped in such biodiscovery programmes, where metataxonomic data collected for community analysis could guide future drug discovery [24,25].

In this study, we investigate the microbiome of 23 diverse sponges from 5 underexplored deep-water seamounts in the Atlantic Ocean. We focus on elucidating the impact of host-sponge class on microbial composition, using refined taxonomic assignments to better profile these communities. We investigate the factors shaping these microbial communities, including the biogeographical factors, and sponge host phylogeny. This study adds novel insight into host phylogeny’s impact on the sponge microbiome and explores previously unstudied sponge taxa from the deep Atlantic Ocean.

## Methods

### Sample selection

**Fig. 1.**
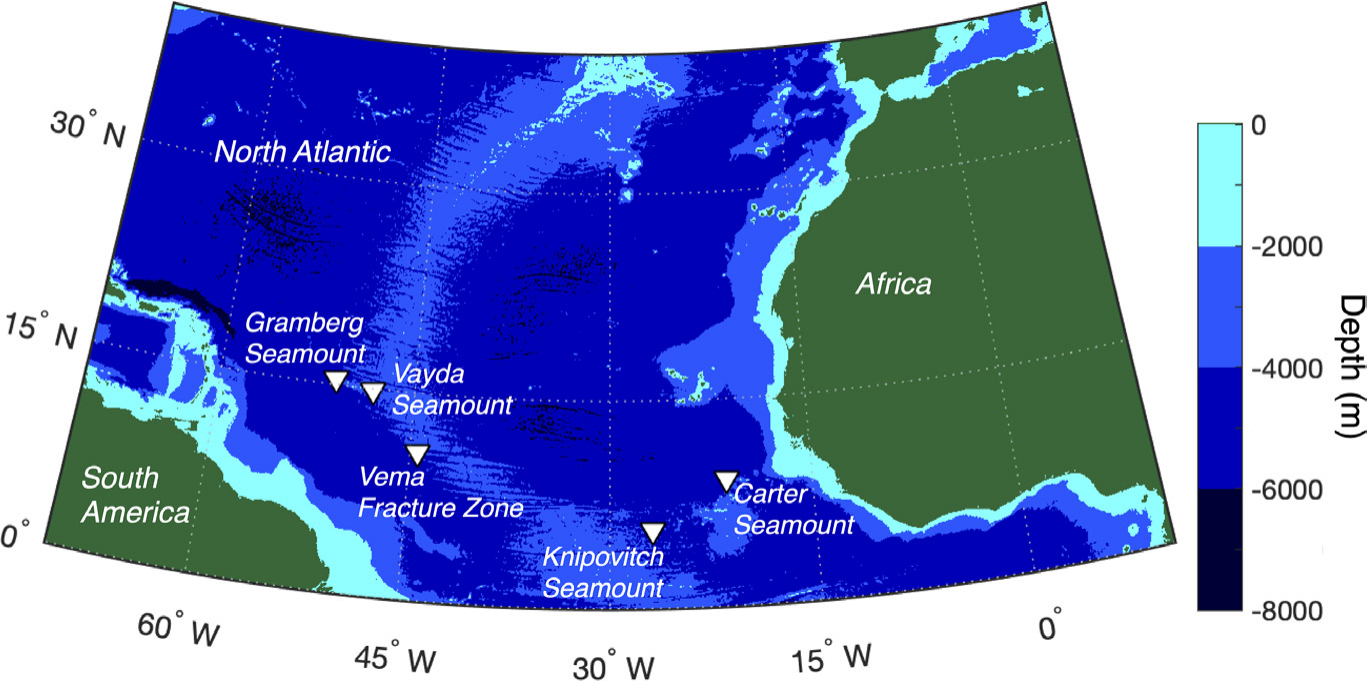
Map of the five deep-sea sampling areas (triangles) from the JC094 research cruise in the mid-Atlantic Ocean. Graphic created using ETOPO1 bathymetry.

A subset of 23 deep-sea sponges was chosen from a larger collection, which was described previously [26]. Sponges were initially selected based on preliminary morphological assessments to aim for a balanced representation of demosponge and hexactinellid samples. Sponges were also selectively sampled to cover a range of depths and geographic distributions across the five sampling sites (Fig. 1). All chosen sponge samples were collected between depths of 569 and 2618 m from five different sampling sites in the Atlantic Ocean by the NERC research vessel RRS James Cook during research cruise JC094 [27]. Samples were provided to us directly by the cruise Chief Scientist Laura Robinson, University of Bristol. Sample handling and preparation were performed according to the protocols adopted for the Earth Microbiome Project (EMP) [28] and subsequently the Sponge Microbiome Project [5]. Local seawater controls were not available for sequencing and so are absent from the dataset.

### DNA extraction

DNA was extracted from 0.25 g of sponge tissue using the DNeasy PowerSoil Kit (Qiagen, Hilden, Germany) using the optimized procedure by Marotz *et al*. [29] and including a blank extraction to account for intrinsic kit or reagent contamination [30]. Prior to extraction, sponge samples were washed three times in sterile artificial seawater (Crystal Sea Marine Mix, Marine Enterprise International). All extractions were performed in a laminar flow hood.

### Identification of sponge species

Sponge taxonomy was assigned based on the mitochondrial cytochrome oxidase subunit I (COI) or the 28S rRNA gene. The COI gene was amplified through PCR using the universal primers LCO1490 and HCO2198 (Table S1, available in the online version of this article) [31]. The reaction comprised 20 µl Platinum Hot Start PCR Master Mix (2X) (Invitrogen, Thermo Fisher Scientific, Waltham, MA, USA), 1.6 µl of each primer at 10 pmol/µl, 14.8 µl deionized water and 2 µl DNA template. Thermal cycling was performed as described by Yang *et al*. [32]: 1 min denaturation at 94 °C; 5 cycles of 94 °C for 30 s, annealing at 45 °C for 90 s and extension at 72 °C for 1 min; 35 cycles of 94 °C for 30 s, 51 °C for 40 s and 72 °C for 1 min and a final extension step at 72 °C for 5 min. Five microlitres of the amplified PCR fragment were analysed on a 1 % Tris-Acetate-EDTA (TAE) agarose gel to confirm the expected COI gene fragment size of approximately 680 bp. Samples unable to be identified by the COI locus were reanalysed with 28S primers NL4F and NL4R (Table S1) [33], using the same PCR reagents but with thermal cycling as follows: 10 min initial denaturation at 95 °C; 35 cycles of 95 °C, 56 °C and 72 °C for 1 min each and a final extension step of 72 °C for 7 min [32]. Five microlitres of these reactions were analysed on a 1 % TAE agarose gel to confirm 28S gene fragment size 858 to 1010 bp. PCR products were then either purified directly using the Monarch PCR and DNA Cleanup Kit (New England Biolabs, Hitchin, UK), or if more than one band was present, extracted from 1 % TAE gel using the QIAquick Gel Extraction Kit (Qiagen, Hilden, Germany). Nucleotide blast (BLASTn) was used for taxonomic assignment [34]. Sponge taxonomy was assigned at the species level if the BLASTn % identity was >97 %, and we recognize that this threshold is arbitrary, but it is sufficient for the purposes of this study [35]. For identities below this threshold or if the closest relative was not named, the assignment was made to the nearest genus, recognizing that lower % identities yield approximate, not precise, taxonomic classifications. Individual sponge samples are referred to by their taxonomic assignment followed by their in-house sample identifier (BXXXX) as a suffix.

### Sponge phylogenetic analysis

COI gene sequences were aligned with MAFFT (v.7.490) using the l-INS-I algorithm [36]. A phylogenetic tree was then constructed using the IQ-TREE2 (v.2.2.2.7) with a model finder, the best-fitting model was TIM2+F+I+G4 and the analysis included 1000 ultra-fast bootstrap replicates [37]. The trimmed COI gene from the slime sponge *Oscarella carmela* (GenBank: NC_009090.1) was used as an outgroup on which the tree was rooted. The tree was visualized and annotated with iTOL v.6 [38].

### Sponge microbiome sequencing

The V4 and V5 regions of the microbial 16S rRNA gene were amplified via PCR with the universal primers 515F-926R [28] combined with Illumina sequencing adaptors (Table S1). Thermal cycling followed the standardized protocol of the EMP [28]. This entailed an initial denaturation at 94 °C for 3 min, followed by 35 cycles of 94 °C for 45 s, 50 °C for 60 s and 72 °C for 90 s, with a final extension of 10 min at 72 °C. The presence of the expected PCR products was confirmed by visualizing 5 µl of the reaction on a 1 % agarose gel. For most sponge samples, this produced a single intense band at approximately 478 bp, and the amplicon pool was cleaned with the QIAquick PCR Purification Kit (Qiagen 28104). If spurious bands were identified, the 478 bp fragment was extracted using the QIAquick Gel Extraction Kit (Qiagen 28704) alongside a blank gel lane extraction as a further negative control for environmental contamination.

Illumina sequencing was performed by the genomic facility at the University of Bristol. After initial quality assessment and normalization of amplicon libraries, samples were indexed with the XT Index Kit (Nextera) and sequenced using the MiSeq platform (Illumina). Base calling and quality assessment were performed with Real-Time Analysis version 1.18.54.0. At least 200000 reads were recovered for each sample.

### Sponge microbiome sequencing and bioinformatic analyses

Microbiome analysis was performed with QIIME2 2023.7 [39]. The data-processing pipeline is available at https://github.com/Sam-Will/Deep_Sponge_Micro. Adaptors and primers were removed from the demultiplexed sequence data using the cutadapt plugin [40], and the subsequent data were denoised with DADA2 (v.1.26) [41] via the denoised-pair command. Truncation parameters were chosen based on the quality plots to ensure a minimum of 30 bp overlap between reads. Amplicon sequence variants (ASVs) were assigned taxonomy using classify-sklearn feature-classifier [42] trained against the Greengenes2 2022.10 full-length sequence classifier [21]. Data were imported to R version 4.0.2 for further analysis [43]. Inherent laboratory contamination during sample handling and DNA extraction was assessed by analysing blank samples from the DNA extraction kit (350 173 sequences) and from the gel extraction kit (398 sequences). Twenty-six of the ASVs in these negative controls were identified as likely true contaminants by the decontam 1.8.0 package in combined mode [44]. These sequences were removed from the sponge dataset (Table S2). Identified contaminants were removed from all samples in the dataset prior to further analysis. Phylogenies were constructed with phyloseq 1.42.0, and the ASV alpha diversity values were calculated using the ‘estimate_richness()’ function and assessed with aov() [43]. Rarefaction curves were calculated with iNEXT (v.2.0.20) [45]. Permutational multivariate analyses of variance (PERMANOVA), non-metric multidimensional scaling (NMDS) and distance decay analyses were calculated using Vegan (v.2.5.7) [46]. Before performing PERMANOVA, the dataset was tested for homoscedasticity using the function betadisper() and then anova(). As homoscedasticity was observed (*P* > 0.05 in ANOVA test), PERMANOVA was then performed on the Bray–Curtis dissimilarity matrix calculated on the Hellinger-transformed ASV dataset (1000 permutations). PERMANOVA was performed using all samples except sample hexactinellid B01175, which was the only representative of >2500-m depth. To calculate the community correlation with sampling depth (i.e. distance decay) and geographical distances, Mantel statistical tests (‘method=Spearman’) were performed on the Bray–Curtis dissimilarity matrix computed on the Hellinger-transformed ASV dataset and Euclidean distance matrix calculated on depth data and geographical distances (1000 permutations). *P*-values obtained from the Mantel tests were corrected using the Holm–Bonferroni method [47]. Geographical distances were calculated using the R library geosphere (v.1.5-1.8) [48]. NMDS was performed on the Hellinger-transformed ASV dataset. Plots and data manipulation were performed using gplots (v.3.1.1), RColorBrewer (v.1.1.2), tidyr (v.1.1.2), ggplot2 (v.3.3.3) and svglite (v.2.1.2). A dendrogram of the sponge microbiome community structure was obtained using the hclust function (‘method=ward.D2’) and was formatted using ape (v.5.7-1) and TreeTools (v.1.10.0) packages [49,50]. All of the above steps were done in the R environment [51]. Dendroscope (v.3.8.10) [52] was to create a tanglegram depicting similarities between the microbiome community structure and sponge phylogeny.

### Data availability

The COI sequences of sponges in this study have been deposited in GenBank with the assigned accession numbers PP092504-PP092519 and OP036683.1. All raw 16S rRNA gene amplicon sequencing data are available in the NCBI Sequence Read Archive, associated with BioProject number PRJNA702029. Additionally, the data-processing pipeline, including QIIME2 taxonomy, representative sequences, phylogenetic trees and metadata, can be found at https://github.com/Sam-Will/Deep_Sponge_Micro.

## Results

### Classification of sponge samples

**Fig. 2.**
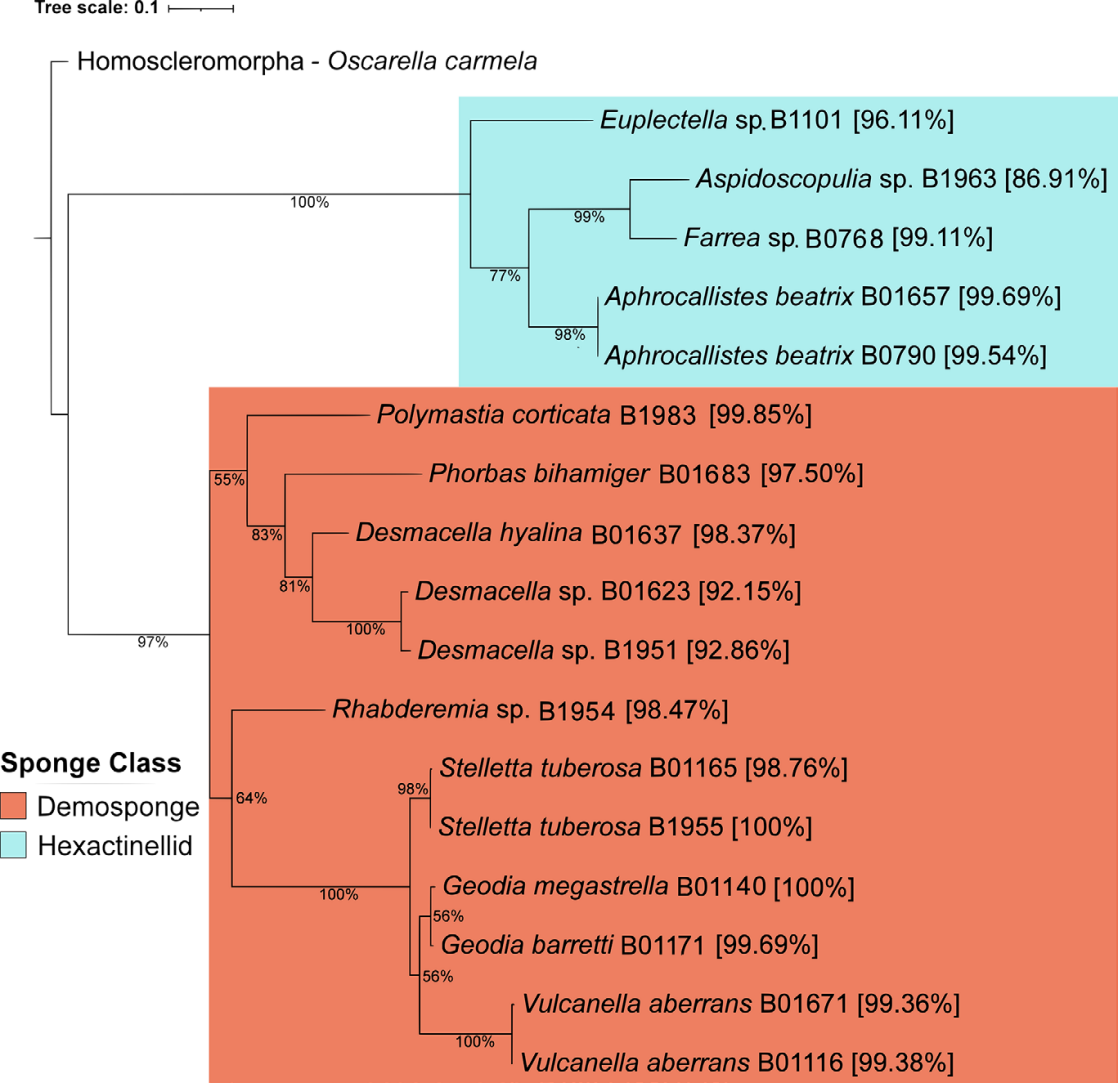
Maximum-likelihood tree for 17 deep-sea sponge samples based on the mitochondrial cytochrome oxidase (COI) gene barcode. Bootstrap values are shown as a percentage of 1000 replications. Taxon branches are labelled with the closest relative from BLASTn (% ID) and the in-house sample identifier. Demospongia are highlighted in orange and Hexactinellida in blue. The slime sponge *O. carmela* COI sequence is used as an outgroup.

Sponge taxonomy was initially based on the gene for mitochondrial cytochrome oxidase [31]. This approach was used to successfully classify 17 of the 23 sponges considered here at the genus or species level (Fig. 2). Assignments for certain samples to the nearest genus, including *Euplectella*, *Desmacella* and *Aspidoscopulia*, are tentative since they have a low blast identity to their nearest relative and thus are likely novel. Of the remaining six sponges, two were subsequently identified to species level by the 28S rRNA gene (*Hertwigia* sp. MD-2008 97.09 % and *Trachycladus* sp. NCI325 97.51 %). The four remaining sponges were identified only to the class level as hexactinellid, based on a visual inspection of spicules. Overall, the 23 deep-sea sponges studied here comprise 13 demosponges with representatives from 6 orders, 8 families and 10 species and 10 hexactinellids from 2 orders, 3 families and 5 species (Fig. 2, Table S3). To our knowledge, this dataset comprises several sponge genera whose microbiota has not previously been characterized, including *Rhabderemia*, *Trachycladus*, *Euplectella* and *Aspidoscopulia* [13,14, 18].

### Microbial ASVs and richness

**Fig. 3.**
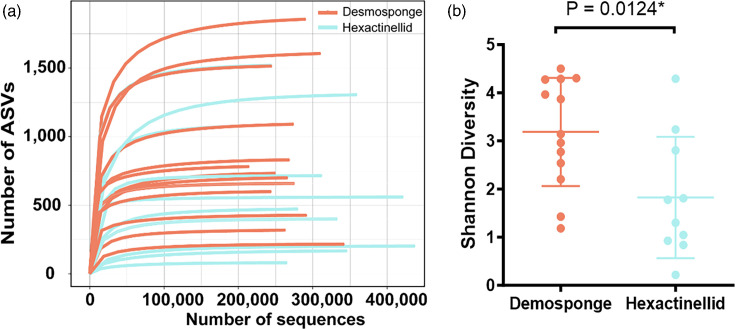
Microbial ASVs and Shannon diversity derived from 23 deep-sea sponges. (**a**) Rarefaction curves of 16S rRNA gene diversity for deep-sea Atlantic sponge samples displayed by sponge class. Results from the hexactinellid *Euplectella* sp. B1101 (1534 ASVs) and the demosponge *Vulcanella aberrans* B0116 (1530 ASVs) superimpose are difficult to distinguish on this plot. (**b**) Shannon diversity associated with each sponge, presented by sponge class.

Sequence abundance from the sponge microbiota for each sponge sample ranged from 200 223 to 437 147, with an average of 292 684 sequences per sponge. While individual sponges varied, across the whole dataset, there was a higher amount of archaeal (3514471) than bacterial abundance (3185734), and for *Hertwigia* sp. B0507 and hexactinellid B01175, archaea represented over 90 % of the reads (Fig. S1). Sequencing depth was sufficiently deep to be confident in recovering an accurate estimate of diversity, with all samples reaching at least 200000 reads after DADA2 denoising (Table S4) and approaching saturation in rarefaction curves (Figs 3a and S2).

The total number of ASVs (i.e. richness) present across the 23 sponges was 11 465. The microbiota of sponge *Desmacella* sp. B1951 had the greatest number of ASVs with 1864, and this was over 20 times the number of ASVs in the least diverse sponge (*Aphrocallistes beatrix* B01657, 80 ASVs; Fig. 3a, Table S5). There was considerably higher bacterial richness at the kingdom level with 10 844 ASVs assigned to bacteria, 595 ASVs assigned to archaea and 26 ASVs that could not be assigned. Hexactinellid samples (*n*=10) had 4698 bacterial and 315 archaeal ASVs, while demosponge samples (*n*=13) collectively had 6753 bacterial and 378 archaeal ASVs. The number of observed ASVs was slightly higher in demosponges compared with hexactinellids, but this difference was not statistically significant (*P*=0.298). Demosponges did, however, exhibit a significantly higher Shannon diversity index (*P*=0.012) (Fig. 3b). The range of ASVs within the dataset was large however, and certain hexactinellid samples from the *Euplectellidae* family (*Euplectella* sp. B1101 and *Hertwigia* sp. B0507) had among the highest number of ASVs (Table S5). Examining the impact of depth on richness and diversity, no correlation was observed between increasing depth on observed ASVs (*P*=0.189) or Shannon diversity (*P*=0.762). However, a relationship emerged between depth and archaeal ASVs in demosponges, with a decrease in the number of observed archaea as depth increased (*P*=0.002).

### Taxonomic distribution and relative abundance of the sponge microbiota

**Fig. 4.**
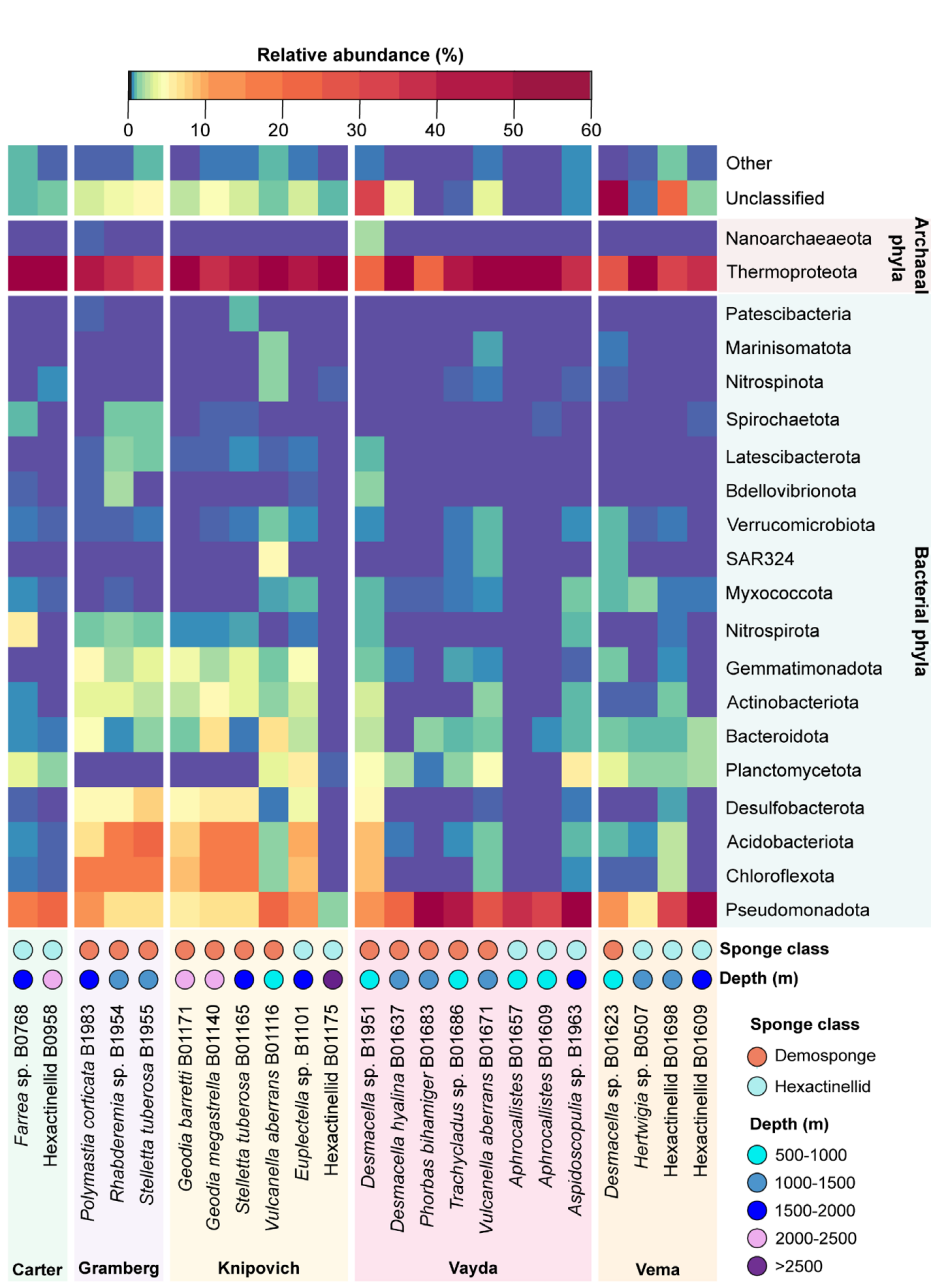
Abundant phyla in the deep-sea sponge microbiome, grouped by sampling location. Only phyla present with a relative abundance >0.5 % of total taxa in at least one sample are reported.

We identified a total of 41 bacterial phyla and 4 archaeal phyla across the 23 sponge species analysed (Fig. 4). There were eight phyla found across all sponges in the dataset: Thermoproteota, Pseudomonadota, Chloroflexota, Desulfobacterota, Bacteroidota, Actinobacteriota, Gemmatimonadota and Myxococcota (File S1). Bdellovibrionota was present in low abundance in all samples except *Desmacella hyalina* where it was not found. Acidobacteria, a dominant phylum in certain sponges, was present in all sponges except for *A. beatrix* B01657. There were a significant amount of reads unclassified even at the phylum level. This was particularly prominent in the two novel *Desmacella* sponge species, which had 56.11 % (sp. B01623) and 32.16 % (sp. B1951) of the relative abundance from unclassified reads. The diversity of phyla hosted varied across species, with *A. beatrix* B01657 hosting as few as 13 phyla, while *V. aberrans* B01671 exhibited a rich diversity of 38 phyla.

Archaea were more relatively abundant than bacteria, with most archaeal abundance represented by phylum Thermoproteota (99.78 %). This came almost completely from the Nitrosopumilaceae family (99.73 %), which were by far the most abundant single taxa in our samples, where the second and third most abundant were family CG1-02-33-12 (Nanoarchaeota phylum) and Thalassarchaeaceae (Thermoplasmatota phylum) with 0.11 and 0.04 %. The most abundant phyla within bacteria were Pseudomonadota (51.4 %), particularly the classes Alpha- and Gammaproteobacteria, although Alphaproteobacteria was absent in both samples of *A. beatrix* obtained from the Vayda Seamount. The sponge-associated Poribacteria phylum was notably scarce among our deep-sea samples but was identified in five demosponge samples and two hexactinellids. When present, this phylum comprised a tiny fraction of the overall diversity (0.0031 % overall), falling beneath the >0.5 % relative abundance threshold plotted in Fig. 4.

### Sponge species and geographic influence on the deep-sponge microbiome

To determine the influence of biotic and abiotic factors on the sponge microbiome, we performed the Hellinger transformation on the ASV dataset and conducted a PERMANOVA analysis using three factors: sponge taxonomic class, sampling site and sampling depth category. The results suggested that sponge class only explained 9 % of variation in the microbiome, whereas the sampling site and the depth category were responsible for 32 and 16 % of the total variation, respectively (Table 1). Mantel tests showed a significant correlation (*P*=0.013) between the ASV dataset and sponge depth (*r*=0.223) but not with geographic distances (*r*=0.031, *P*=0.302). While geographical distance does not affect microbiome composition, sampling site and depth do have significant impacts, highlighting the influence of environmental locality on community structure [53].

**Table 1.** PERMANOVA (A) and Mantel (B) tests performed on the Hellinger-transformed ASV dataset, using the Bray–Curtis dissimilarity matrix with 1000 permutations

		Factor	*R* ^2^	*P*
A	PERMANOVA	Sampling site	0.319	0.001
		Depth category*	0.164	0.003
		Sponge class	0.085	0.002
		Sampling site: depth category*	0.211	0.009

		Factor	*r*	*P*†
B	Mantel test	Geographical distance	0.031	0.302
		Depth	0.223	0.013

* Depth categories as shown in Fig. 4.

† Adjusted *P*-values using the Holm–Bonferroni method.

Next, we conducted an NMDS analysis of the community composition against the same factors of sponge class, site and depth (our dataset was insufficient for within-species comparisons). The NMDS groupings by sponge class were overlapping to some degree, although they could be distinguished (Fig. S3a). The sampling site and depth did not seem to be associated with clearly distinct clusters (Fig. S3b, c). The most obvious individual cluster for the sampling site was formed by the three demosponge samples from Gramberg Seamount (Fig. S3b). Collectively, while PERMANOVA revealed that site and depth were significant factors in explaining variation in microbial communities, our limited dataset did not show clear and obvious distinctions in community composition when comparing at the finer-grained level of sites or specific depths.

**Fig. 5.**
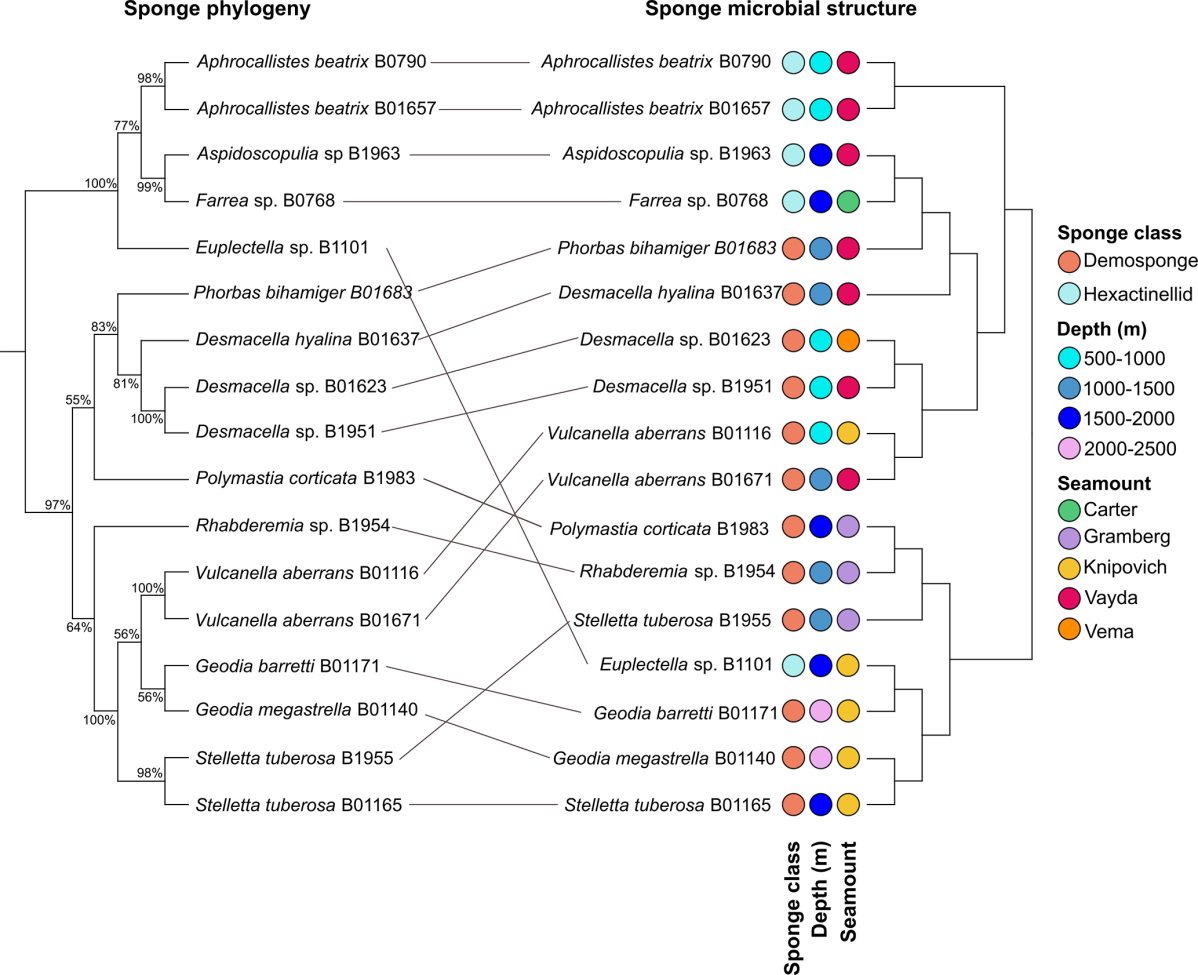
The association between sponge host phylogeny based on COI gene sequences and associated microbial community structure. The left tree represents the COI gene-based phylogenetic relationships among sampled sponge species, while the right tree illustrates the phylogenetic structure of their respective microbial communities. Lines connecting the two trees highlight the observed congruence, demonstrating the influence of host phylogeny on microbial assemblage.

Finally, while the PERMANOVA analysis highlighted that at the higher taxonomic groupings sponge class explained 9 % of variance, previous studies have suggested that sponge-associated microbial communities exhibit a high degree of host specificity at lower taxonomic ranks [13]. We sought to investigate this phenomenon. To accomplish this, we performed a co-phylogenetic analysis relating the phylogenetic structure of sponge microbial communities to the COI gene-based phylogenetic tree from the host sponges. This comparative analysis showed similar but not identical relationship between the microbial assemblage and the host phylogeny (Fig. 5). Our findings highlight the predominance of host species in shaping the structure of the deep-sea sponge microbiome, mostly irrespective of variations in depth or geographical distribution of the same sponge species. One exception here is represented by the two samples of *Stelletta tuberosa*, where it is clear that variation in depth and site played a larger role. Additionally, an anomaly was noted in the sponge *Euplectella* sp. B1101, which demonstrated a phylogenetic affinity more closely aligned with *Geodia* sponges than with other members of the Hexactinellida.

## Discussion

The microbial ecology of the deep-sea remains largely underexplored. This study employs metataxonomic methods to investigate the microbial community structure of 23 sponges from 5 Atlantic seamounts, including many sponges that have not previously been characterized and are likely to be unique to the deep ocean. We reveal a microbiome predominantly consisting of ammonia-oxidizing archaea from the family Nitrosopumilaceae. Importantly, our data provide further evidence that host-sponge phylogeny is associated with microbiome composition, contributing to a further understanding of deep-sea sponge microbial ecology.

## Nitrosopumilaceae dominate the deep-sea sponge microbiome

Our findings corroborate prior observations of the high abundance of archaea, particularly the family Nitrosopumilaceae from the Thermoproteota phylum [8,14,16]. Metagenome-assembled genomes of sponge-associated Nitrosopumilaceae show a high degree of symbiotic adaption [54]. In our study, an overwhelming 99.73 % of all archaeal reads were identified as Nitrosopumilaceae, underscoring their key role in oxidizing ammonia, and so contributing to the process of nitrogen fixation within the sponge microbiome [6,55]. To contextualize the high relative abundance of archaea, predominantly Nitrosopumilaceae, we reanalysed data from a comprehensive dataset collected from predominantly shallow-water sponges [12]. In this analysis, we found that only 6 % of relative abundance could be attributed to archaea compared with over half in our dataset. Symbiotic Nitrosopumilaceae genomes also contain genes involved in the 3-hydroxypropionate/4-hydroxybutyrate cycle, an autotrophic pathway for carbon fixation. In the deep sea, where carbon fixation through photosynthetic pathways in Cyanobacteriota is absent, Nitrosopumilaceae may fulfil an important dual role in ammonia oxidization and carbon fixation [6,7, 56].

## Methodological limitations

Even though we adopted stringent sample rinsing protocols, seawater contamination remains a concern, as do reads present in the blank extraction. ASV richness per sponge is likely slightly elevated due to a lack of seawater controls [13]; to address these concerns, we performed a supplementary analysis of amplicon data from deep-sea seawater collected by Busch *et al*. [13]. We focused on the most abundant genera from these data and confirmed that only three genera appeared at comparable relative abundance across both datasets: two genera from the Pseudomonadota family, Alteromonadaceae (*Pseudoalteromonas* and *Alteromonas*) and the *Roseibacillus* genus from the Verrucomicrobiota phylum. While these findings suggest these groups are potential seawater contaminants, the exclusion of these genera during the PERMANOVA analysis shows no change to the results, supporting the conclusion and validity of our findings (Table S6). The sponges studied here are a subset of a larger collection collected to study biomineralization [57] and subsequently bioprospecting [26,58, 59]. The samples thus represent an unbiased general survey, with a modest sample size, rather than a systematic effort to understand a particular sponge species or geographical location. Consequently, it is important to acknowledge the limitation of drawing any species-level microbiome conclusions from single biological samples. For these analyses, we direct readers to large systematic studies of the deep-sea sponge microbiome [13,14].

## Comparison with large-scale studies

Our findings align with Busch *et al*.’s extensive study of 1077 deep-sea sponges [13] but add novel, previously unstudied genera of deep-sea sponges. The use of the Greengenes2 reference tree improves microbial taxonomic assignment and facilitates comparison with the Genome Taxonomy Database [21,60]. Our study also confirms the presence of both LMA and HMA sponges in deep-sea environments [61]. Interestingly, while hexactinellids generally appear to be LMA, we do identify a potential HMA hexactinellid class sponge from the genus *Euplectella*, a genus that has not been characterized previously and has a microbiome similar to phylogenetically distance sponges. Busch *et al*. noted that *Amphidiscella caledonica* (family *Euplectellidae*) diverged from typical LMA glass sponges, showing high Chloroflexota abundance, and several other members of *Euplectellidae* family had microbiomes more similar to demosponges than other hexactinellids. We identify a further *Euplectellidae* genus (*Euplectella*), which deviates from the classical low Chloroflexota composition of LMA glass sponges [11].

## Genus-specific comparisons

Our study also includes two representatives of the demosponge genus *Geodia* collected from the Knipovich Seamount. Both sponges have similar community profiles to samples of *Geodia hentscheli* and *Geodia barretti* from other studies [8,18, 62], being dominated by Pseudomonadota, Chloroflexota and Acidobacteriota, as well as Desulfobacterota and Gemmatimonadota. These other studies did report a higher abundance of Poribacteria that was not observed here. Otherwise, samples of *S. tuberosa* from Gramberg and Knipovich Seamounts had relatively dissimilar profiles based on the tanglegram analysis (Fig. 5), whereas the two *V. aberrans* samples from Knipovich and Vayda sites clustered together strongly. The relative dominance of Chloroflexota and Acidobacteriota in *S. tuberosa* was previously observed in another member of the genus, *Stelletta normani*, obtained from canyons of the North Atlantic and is consistent with the assignment of this species as a deep-sea HMA sponge [15].

## Influence of sponge host and biogeographical factors

Deep-sea sponge microbial communities are influenced by the sponge host species and biogeographical factors such as nutrient profile and the surrounding seawater [18]. Our study emphasizes the role of depth in shaping sponge-associated communities, a finding that is supported by Busch *et al*. [13] and the *Tara* Oceans project [63], but contrasting with other systematic studies [14]. A study of the impact of seamounts on the microbial composition of sponges highlighted the influence of depth on phyla distribution [18], suggesting that depth could affect communities in an environment-specific manner. The deep-sea Sponge Microbiome Project highlighted a weak but significant biogeographical impact on community composition, albeit over a much larger distance of 10 000 km [13]. In our study, significant differences between sponges at various sites suggest that isolation by seamounts, rather than geographical distance, influences community structure. The influence of the sampling site over geographical distance has been previously observed in deep-sea microbiomes both in sponge hosts and in deep-sea sediments [53,64].

We also identified the accordance between sponge host phylogeny and sponge microbiome structure (Fig. 5), indicating host-driven microbiome composition. For example, the microbiomes of two *A. beatrix* sponges, both collected from the Vayda Seamount, displayed a consistent microbial community, also supporting previous findings from the same species [14]. The identification of a co-phylogenetic relationship, known as phylosymbiosis, has been previously identified in coral [65], mammals, insects [66] and shallow reef sponge microbiomes [67]. While our findings indicate an influence of host phylogeny on microbiome structure, this relationship is not absolute, reflecting that deep-sea sponges may prioritize functional redundancy over species specificity in their microbial associations. This is supported by a comparative study of 39 HMA sponge species, revealing a more pronounced species specificity in microbial composition among shallow-water sponges than in their deep-sea counterparts [68].

## Conclusion

Overall, our study explores the microbiomes of previously unstudied deep-sea sponges and identifies depth and sponge phylogeny to be drivers of microbial composition. Our results particularly highlight distinct microbiomes in the *Euplectellidae* family, differing from other glass sponges, that warrant further investigation.

## supplementary material

10.1099/mic.0.001478 Uncited Supplementary Material 1.

## Abbreviations

ASV: amplicon sequence variant
COI: cytochrome oxidase subunit I
EMP: Earth Microbiome Project
HMA: high microbial abundance
LMA: low microbial abundance
NMDS: non-metric multidimensional scaling
PERMANOVA: permutational multivariate analyses of variance
rRNA: ribosomal RNA
TAE: Tris-acetate-EDTA
