# Supplementary material for: Diversity and structure of the deep-sea sponge microbiome in the equatorial Atlantic Ocean
Source: Microbiology (Reading). 2024 Jul 29;170(7):001478. doi: 10.1099/mic.0.001478 (PMC11286294; doi:10.1099/mic.0.001478)
Supplement: Uncited Supplementary Material 1. [file mic-170-01478-s001.pdf]

Supplementary materials

Diversity and structure of the deep-sea sponge microbiome in the equatorial Atlantic Ocean

Sam E. Williams, Gilda Varliero, Miguel Lurgi, James EM. Stach, Paul R. Race and Paul Curnow

Table of Contents

1) **Supplementary Figures** **2-4**

Figure S1: Archaea and Bacteria Relative Abundance in Sponge Microbiome

Figure S2: 16S rRNA Gene Diversity Rarefaction in Atlantic Sponges

Figure S3: NMDS Analysis of Sponge Microbiome Compositional Similarity

2) **Supplementary Tables** **5-9**

Table S1: PCR Primer Sequences and Target Genes

Table S2: Identified and Removed Contaminants in DADA2 ASV

Table S3: Taxonomic Assignment, Sampling Site, and Depth of Sponges

Table S4: DADA2 Denoising in Sponge Sample Sequencing

Table S5: Alpha Diversity Metrics in Deep-Sea Sponge Samples

Table S6: PERMANOVO Analysis Excluding Sea-Water Taxa

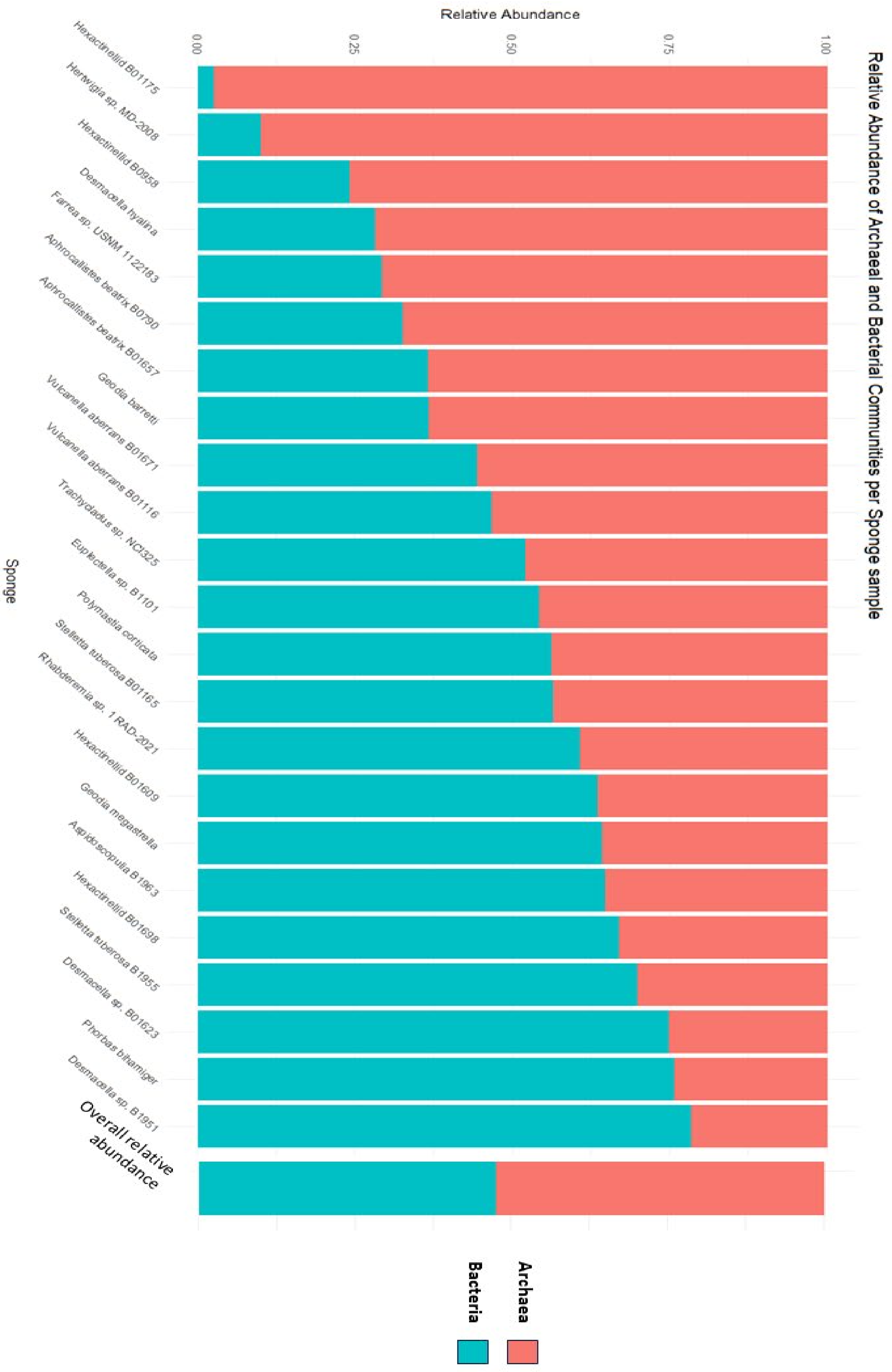

Figure S1. Relative abundance of Archaea and Bacteria in the deep-sea sponge microbiome. Overall relative abundance is taken from the entire dataset.

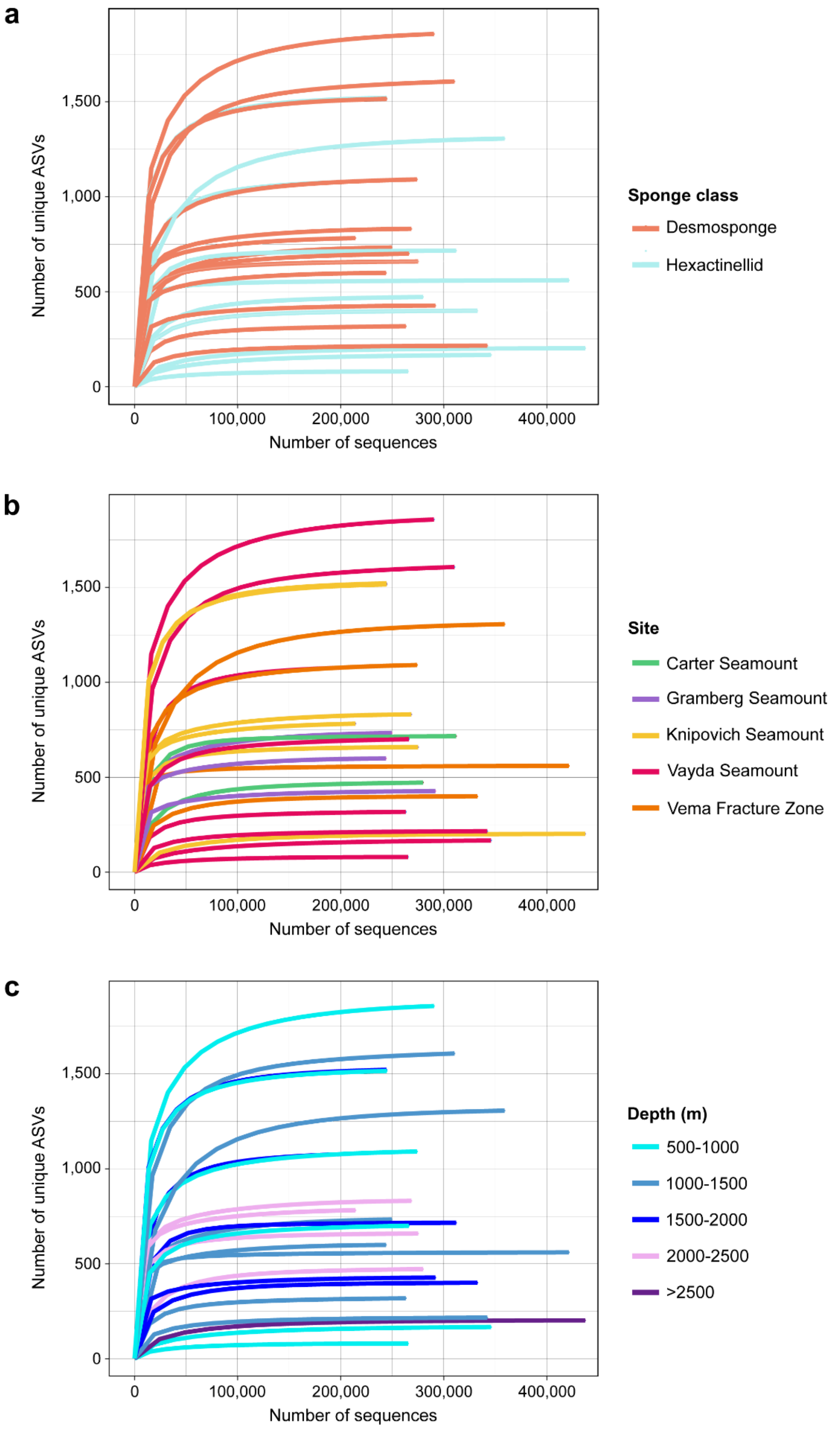

Figure S2. Rarefaction curves of 16S rRNA gene diversity for deep-sea Atlantic sponge samples. Displayed by a) sponge class b) site and c) depth.

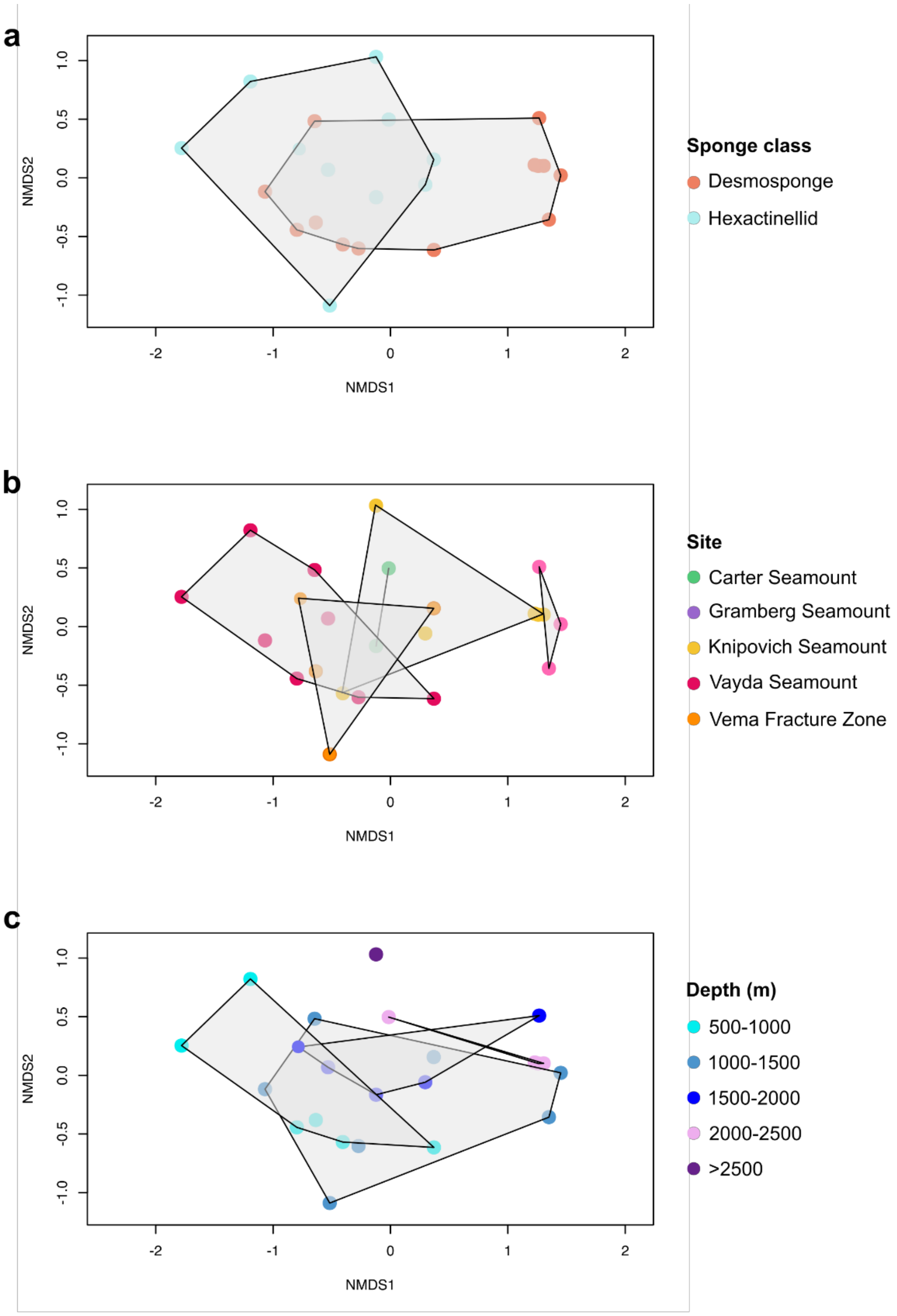

Figure S3. Compositional similarity of the deep-sea sponge microbiome in the Atlantic Ocean. NMDS performed on the Hellinger-transformed ASV dataset. Samples are grouped by (a) sponge class, (b) sampling site and (c) sampling depth.

Table S1. PCR primer sequences used in the study including the target gene and the nucleotide sequence.

Primer name	Target gene	Sequence	Reference
515F	16S rRNA	5′-GTG YCA GCM GCC GCG GTA A-3′	(1)
926R	16S rRNA	5′-CCG YCA ATT YMT TTR AGT TT-3′	(1)
Illumina-515F	16S rRNA	5′-TCG TCG GCA GCG TCA GAT GTG TAT AAG AGA CAG GTG YCA GCM GCC GCG GTA A-3′	(2)
Illumina-926R	16S rRNA	5′-GTC TCG TGG GCT CGG AGA TGT GTA TAA GAG ACA GCC GYC AAT TYM TTT RAG TTT-3′	(2)
LCO1490	COI gene	5′-GGT CAA CAA ATC ATA AAG ATA TTG G-3′	(3)
HCO2198	COI gene	5′-TAA ACT TCA GGG TGA CCA AAA AAT CA-3	(3)
NL4F	28S rRNA	5′-GAC CCG AAA GAT GGT GAA CTA-3′	(4)
NL4R	28S rRNA	5′-ACC TTG GAG ACC TGA TGC G-3′	(4)

Table S2. DADA2 ASV features identified by decontam 1.8.0 as contaminants which were then removed from the final data set

DADA2_Contaminant_Feature_ID	Taxonomic Assignment
50633c4b837aa1a3b21742c0765270a4	Unassigned;NA;NA;NA;NA;NA;NA
ec1735efd303dce8c3a0043d92ff93c8	D_0__Bacteria;D_1__Acidobacteria;D_2__Subgroup 26;NA;NA;NA;NA
15157ac5595c109ea66f313a3eb5eb7a	D_0__Archaea;D_1__Thaumarchaeota;D_2__Nitrososphaeria;D_3__Nitrosopumilales;D_4__Nitrosopumilaceae;NA;NA
f21dfad54cde3762dea1d00a41729507	D_0__Archaea;NA;NA;NA;NA;NA;NA
d838a3400b85af80da105b0545a6c789	D_0__Bacteria;D_1__Planctomycetes;D_2__Planctomycetacia;D_3__Pirellulales;D_4__Pirellulaceae;D_5__uncultured;NA
d0bffe0ce5e1b758547fe2b490066b94	D_0__Bacteria;D_1__Planctomycetes;D_2__Planctomycetacia;D_3__Pirellulales;D_4__Pirellulaceae;NA;NA
f1be6b0c6e4b3136ead03bc4d5c67c79	D_0__Bacteria;D_1__Acidobacteria;D_2__Subgroup 6;D_3__uncultured bacterium AD106-G4;D_4__uncultured bacterium AD106-G4;D_5__uncultured bacterium AD106-G4;D_6__uncultured bacterium AD106-G4
b9b990ccd54fd7edbe52563acac38b90	D_0__Bacteria;D_1__Acidobacteria;D_2__Subgroup 6;NA;NA;NA;NA
a04b27b43cec356541b84c9346d36125	D_0__Bacteria;D_1__Chloroflexi;D_2__Dehalococcoidia;D_3__SAR202 clade;NA;NA;NA
63eed8bec991106ef19f974d5f2063f2	D_0__Bacteria;D_1__Chloroflexi;D_2__Dehalococcoidia;D_3__SAR202 clade;D_4__uncultured Chloroflexus sp.;D_5__uncultured Chloroflexus sp.;D_6__uncultured Chloroflexus sp.
c055aacdfae9d0169fc1c6f884292c4c	D_0__Bacteria;D_1__Deinococcus-Thermus;D_2__Deinococci;D_3__Thermales;D_4__Thermaceae;D_5__Meiothermus;D_6__uncultured organism
3464a7e5d5c33c0a88af22e879eb89a4	D_0__Bacteria;D_1__Proteobacteria;D_2__Deltaproteobacteria;D_3__Desulfarculales;D_4__Desulfarculaceae;D_5__uncultured;D_6__uncultured delta proteobacterium
d2bad679382dfdf5b82f911661bf5dfb	D_0__Bacteria;D_1__Proteobacteria;D_2__Gammaproteobacteria;D_3__KI89A clade;D_4__uncultured bacterium;D_5__uncultured bacterium;D_6__uncultured bacterium
dd606ddbf01aa42616b1135ac87f3fea	D_0__Bacteria;D_1__Proteobacteria;D_2__Gammaproteobacteria;D_3__Alteromonadales;D_4__Moritellaceae;D_5__Moritella;NA
0d53eae08491290ff50a3c98788baf5b	D_0__Bacteria;D_1__Proteobacteria;D_2__Gammaproteobacteria;D_3__P0X4b2H11;D_4__uncultured bacterium;D_5__uncultured bacterium;D_6__uncultured bacterium
5921c63ef5ace7407df7613b0113ea46	D_0__Bacteria;D_1__Proteobacteria;D_2__Gammaproteobacteria;D_3__Betaproteobacteriales;D_4__Methylophilaceae;D_5__OM43 clade;NA
927ef3265e9e3355963d4408200e31f8	D_0__Bacteria;D_1__Actinobacteria;D_2__Actinobacteria;D_3__Micrococcales;D_4__Micrococcaceae;D_5__Micrococcus;NA

Table S3. Taxonomic assignment of sponges, sampling site and depth information. Method of identification and BLASTn % identity to closest relative

Class	Order	Family	Genus and species (Closest relative on NCBI GenBank)	Name in this study	Samples	Sampling location	Sampling depths (m)	Method of identification (BLASTN %)
Demosponge	Desmacellida	*Desmacellidae*	*Desmacella cf. annexa*	*Desmacella sp. B1951, Desmacella sp. B01623*	2	Vema, Vayda	710, 926	CO1 (92.86, 92.15%)
Demosponge	Desmacellida	*Desmacellidae*	*Desmacella hyalina*	*Desmacella hyalina* B01637	1	Vayda	1153	CO1 (98.37%)
Demosponge	Poecilosclerida	*Hymedesmiidae*	*Phorbas bihamiger*	*Phorbas bihamiger* B01683	1	Vayda	1483	CO1 (97.50%)
Demosponge	Biemnida	*Rhabderemiidae*	*Rhabderemia sp. 1 RAD-2021*	*Rhabderemia sp.* B1954	1	Gramberg	1127	CO1 (98.47%)
Demosponge	Polymastiidae	*Polymastiidae*	*Polymastia corticata*	*Polymastia corticata* B1983	1	Gramberg	1869	CO1 (99.85%)
Demosponge	Tetractinellida	*Vulcanellidae*	*Vulcanella aberrans*	*Vulcanella aberrans B01671, Vulcanella aberrans B01116*	2	Vayda, Knipovich	1150, 701	CO1 (99.36%, 99.38%)
Demosponge	Tetractinellida	*Geodiidae*	*Geodia barretti*	*Geodia barretti* B01171	1	Knipovich	2257	CO1 (99.69%)
Demosponge	Tetractinellida	*Geodiidae*	*Geodia megastrella*	*Geodia megastrella* B01140	1	Knipovich	2307	CO1 (100%)
Demosponge	Tetractinellida	*Ancorinidae*	*Stelletta tuberosa*	*Stelletta tuberosa B1955, Stelletta tuberosa B01165*	2	Gramberg, Knipovich	1460, 2307	CO1 (100%, 98.76%)
Demosponge	Trachycladida	*Trachycladidae*	*Trachycladus sp. NCI325*	*Trachycladus sp.* B01686	1	Vayda	569	28S (97.51%)
Hexactinellid	Sceptrulophora	*Aphrocallistidae*	*Aphrocallistes beatrix*	*Aphrocallistes beatrix B0790, Aphrocallistes beatrix B01657*	2	Vayda	742, 865	CO1 (99.54, 99.69%)
Hexactinellid	Lyssacinosida	*Euplectellidae*	*Hertwigia sp. MD-2008*	*Hertwigia sp.* B0507	1	Vema	1140	28S (97.09%)
Hexactinellid	Sceptrulophora	*Farreidae*	*Farrea sp. USNM 1122183*	*Farrea sp.* B0768	1	Carter	1544	CO1 (99.11%)
Hexactinellid	Lyssacinosida	*Euplectellidae*	*Euplecterlla sp. HBOI*	*Euplecterlla sp. B1101*	1	Knipovich	1505	CO1 (96.11%)
Hexactinellid	Sceptrulophora	*Farreidae*	*Aspidoscopulia ospreya*	*Aspidoscopulia sp. B1963*	1	Vayda	1706	CO1 (86.91%)
Hexactinellid	NA	NA	NA	NA	4	Vema, Carter, Knipovich	1302, 1648, 2318, 2618	Spicule morphology

Table S4. DADA2 denoising of raw sequencing to amplicon sequence variants for each sponge sample and two negative controls

Sample-code	*Sample-ID*	*input*	*filtered*	*denoised*	*merged*	*non-chimeric*
B0958	Hexactinellid B0958	362870	306353	306353	293968	279823
B0507	*Hertwigia* sp. B0507	454548	402398	402398	392163	359254
B01671	*Vulcanella aberrans* B01671	451350	370365	370365	341796	310478
B01165	*Stelletta tuberosa* B01165	411178	355792	355792	303745	214299
B1101	*Euplectella sp.* B1101	346965	281666	281666	262303	244673
B01637	*Desmacella hyalina* B01637	326105	276846	276846	271328	263138
B0768	*Farrea* sp. B0768	403221	326306	326306	323577	312162
B01623	*Desmacella sp.* B01623	408675	353907	353907	328635	274158
B01171	*Geodia barretti* B01171	397309	334193	334193	300019	275125
B1951	*Desmacella sp.* B1951	446100	370664	370664	327932	291343
B1963	*Aspidoscopulia sp.* B1963	317991	256963	256963	248992	200223
B01657	*Aphrocallistes beatrix* B01657	304879	272175	272175	269622	265216
B01116	*Vulcanella aberrans* B01116	433479	356167	356167	331611	244735
B0790	*Aphrocallistes beatrix* B0790	419338	362530	362530	354738	345848
B01683	*Phorbas bihamiger* B01683	455059	358571	358571	355041	342180
B01609	Hexactinellid B01609	424806	375249	375249	370658	332808
B01175	Hexactinellid B01175	512952	456317	456317	454292	437157
B01686	*Trachycladus* sp. B01686	402187	334188	334188	317666	266038
B1954	*Rhabderemia* sp. B1954	400168	345122	345122	288800	251398
B1955	*Stelletta tuberosa* B1955	474722	374922	374922	309185	244069
B01140	*Geodia megastrella* B01140	498304	415785	415785	349302	268611
B01698	Hexactinellid B01698	564951	435208	435208	432029	423233
B1983	*Polymastia corticata* B1983	401621	346304	346304	319266	291842
Blank	-	429850	364245	364245	355614	350173
Gel-extract-neg	-	2269	662	662	462	398

Table S5. Alpha diversity metrics of each deep-sea sponge sample ordered by Observed ASVs.

Sponge sample	Observed	Chao1	Shannon	InvSimpson
Desmacella sp. B1951	1864	1887.90	4.50	11.35
Vulcanella aberrans B01671	1610	1644.71	2.77	3.55
Euplectella sp. B1101	1534	1552.55	4.29	7.97
Vulcanella aberrans B01116	1530	1537.48	4.29	9.52
Hertwigia sp. B0507	1271	1294.95	1.30	1.44
Desmacella sp. B01623	1098	1109.23	2.96	3.32
Aspidoscopulia sp. B1963	1086	1102.60	2.80	4.33
Geodia megastrella B01140	851	853.76	4.30	19.17
Stelletta tuberosa B01165	784	788.36	4.28	16.75
Rhabderemia sp. B1954	739	741.25	3.87	6.80
Farrea sp. B0768	734	736.15	1.81	2.13
Trachycladus sp. B01686	706	710.50	2.20	3.35
Geodia barretti B01171	669	670.06	2.54	2.63
Stelletta tuberosa B1955	595	595.61	3.96	10.67
Hexactinellid B01698	566	568.14	3.24	8.48
Hexactinellid B0958	481	495.44	1.05	1.69
Polymastia corticata B1983	439	440.00	3.14	5.34
Hexactinellid B01609	411	412.27	1.78	3.19
Desmacella hyalina B01637	312	315.06	1.18	1.92
Phorbas bihamiger B01683	216	218.00	1.43	3.22
Hexactinellid B01175	202	202.79	0.22	1.06
Aphrocallistes beatrix B0790	150	151.00	0.93	1.94
Aphrocallistes beatrix B01657	80	80.43	0.84	1.99

Table S6. PERMANOVA analysis following removal of potential seawater contaminants from: *Pseudoalteromonas and Alteromonas* and the *Roseibacillus*. *Significance increased without a change in R^2

	Factor	R^2	*p*
PERMANOVA	Sampling Site	0.319	0.001
	Depth Category	0.164	0.002*
	Sponge Class	0.085	0.002
	Sampling Site: Depth Category	0.211	0.005*

References:

1. Parada AE, Needham DM, Fuhrman JA. Every base matters: assessing small subunit rRNA primers for marine microbiomes with mock communities, time series and global field samples. Environ Microbiol. 2016;18(5):1403-14.
2. Thompson LR, Sanders JG, McDonald D, Amir A, Ladau J, Locey KJ, et al. A communal catalogue reveals Earth's multiscale microbial diversity. Nature. 2017.
3. Folmer O, Black M, Hoeh W, Lutz R, Vrijenhoek R. DNA primers for amplification of mitochondrial cytochrome c oxidase subunit I from diverse metazoan invertebrates. Mol Mar Biol Biotechnol. 1994;3(5):294-9.
4. Nichols SA. An evaluation of support for order-level monophyly and interrelationships within the class Demospongiae using partial data from the large subunit rDNA and cytochrome oxidase subunit I. Molecular Phylogenetics and Evolution. 2005;34(1):81-96.
